# 670nm photobiomodulation modulates bioenergetics and oxidative stress, in rat Müller cells challenged with high glucose

**DOI:** 10.1371/journal.pone.0260968

**Published:** 2021-12-03

**Authors:** Hannah J. Nonarath, Alexandria E. Hall, Gopika SenthilKumar, Betsy Abroe, Janis T. Eells, Elizabeth S. Liedhegner

**Affiliations:** Department of Biomedical Sciences, College of Health Sciences, University of Wisconsin-Milwaukee, Milwaukee, Wisconsin, United States of America; Save Sight Institute, AUSTRALIA

## Abstract

Diabetic retinopathy (DR), the most common complication of diabetes mellitus, is associated with oxidative stress, nuclear factor-κB (NFκB) activation, and excess production of vascular endothelial growth factor (VEGF) and intracellular adhesion molecule-1 (ICAM-1). Muller glial cells, spanning the entirety of the retina, are involved in DR inflammation. Mitigation of DR pathology currently occurs via invasive, frequently ineffective therapies which can cause adverse effects. The application of far-red to near-infrared (NIR) light (630-1000nm) reduces oxidative stress and inflammation *in vitro* and *in vivo*. Thus, we hypothesize that 670nm light treatment will diminish oxidative stress preventing downstream inflammatory mechanisms associated with DR initiated by Muller cells. In this study, we used an *in vitro* model system of rat Müller glial cells grown under normal (5 mM) or high (25 mM) glucose conditions and treated with a 670 nm light emitting diode array (LED) (4.5 J/cm^2^) or no light (sham) daily. We report that a single 670 nm light treatment diminished reactive oxygen species (ROS) production and preserved mitochondrial integrity in this *in vitro* model of early DR. Furthermore, treatment for 3 days in culture reduced NFκB activity to levels observed in normal glucose and prevented the subsequent increase in ICAM-1. The ability of 670nm light treatment to prevent early molecular changes in this *in vitro* high glucose model system suggests light treatment could mitigate early deleterious effects modulating inflammatory signaling and diminishing oxidative stress.

## Introduction

Diabetes affects 10% of the population in the United States [1] and causes complications throughout the body due to sustained high circulating glucose which damages the vasculature resulting in chronic macrovascular and microvascular damage [2]. In particular, microvascular damage leads to retinopathy, neuropathy and nephropathy in diabetic patients. Indeed, diabetic retinopathy (DR) has become a leading cause of blindness in the United States [1]. The pathophysiology of DR is complicated, with early pathologic changes including mitochondrial dysfunction, oxidative stress and inflammation [3, 4], and later changes including elevated concentrations of vascular endothelial growth factor (VEGF) and a breakdown of the inner blood-retinal barrier, resulting in the extracellular fluid accumulation in macula and disturbed vision [2, 5–7]. This study is focused on the early pathological changes associated with DR and a potential mitigation strategy with treatment with far-red light, or photobiomodulation (PBM).

PBM is the process by which specific wavelengths of light are absorbed by cellular photoacceptor molecules, resulting in the activation of signaling pathways that culminate in subsequent biological changes within the cell. PBM occurs as a result of low-intensity photochemical reactions in the cell in contrast to thermal photo-ablation produced by high-intensity lasers [8]. PBM has been shown to act on mitochondria-mediated signaling pathways to preserve mitochondrial function, attenuate oxidative stress, stimulate the production of cytoprotective factors, preventing cell death in *in vitro* and *in vivo* experimental models [9–11]. In retinal cell culture and animal models of DR, 670 nm light therapy resulted in decreased reactive oxygen species (ROS) production [12, 13] and slowed disease progression [10, 14, 15]. In a rat model of DR, Tang *et al*. found that 670 nm light reduced retinal ganglion cell death and improved retinal function. Importantly, PBM has been shown to attenuate inflammation and oxidative stress and protect retinal function in experimental animal and human studies [12, 13]. Additionally, PBM is currently being examined as a treatment for diabetic macular edema in a multi-center clinical trial (Clinical trial NCT03866473). The mechanisms by which PBM exerts a beneficial effect have not been clearly elucidated. Studies have shown that 670 nm light is absorbed by cytochrome-c-oxidase promoting mitochondrial bioenergetic function and increasing ATP synthesis [9, 10, 12, 14], however other pathways have been mechanistically implicated including alteration of transcription [10, 16], release of nitric oxide [17, 18], and activation of kinases such as Akt [19, 20]. Regardless of the exact mechanism, studies in both mice and humans have shown improved vision with PBM treatment [11, 13]. The goal of this study is to increase the mechanistic understanding of how 670nm light improves retinal outcomes. We hypothesize that 670nm PBM will mitigate cellular changes caused by high glucose through modulation of ROS and mitochondrial function leading to decreased inflammatory marker production. We tested this hypothesis in an immortalized Müller retinal glial cell model of early DR. These cells were chosen due to the abundance of studies characterizing cellular changes and their impact in response to high glucose [21–28] to allow for understanding of how these changes are modulated by 670nm light in order to characterize a potential mechanism for the beneficial effects seen with 670nm treatment in various models of DR [12, 13, 27, 29–31].

The importance of Müller glial cells in retinal disease is becoming increasingly understood. These cells are the principal glia of the retina, spanning the entire retina with intimate contact of both retinal blood vessels and retinal neurons [23]. Normally, Müller glial cells function to recycle neurotransmitters, retinoic acid compounds, and ions (including potassium), to control of metabolism and supply of nutrients for the retina, and to regulate blood flow and maintenance of the blood retinal barrier [26, 32]. In DR, hyperglycemia-induced retinal injury activates Müller cells leading to a stress response that promotes inflammation and angiogenesis [5, 6, 33, 34]. Thus, Müller glial cells significantly contribute to DR [22, 23, 28, 32–36]. In this study, we show improved cellular changes with 670nm treatment in Müller glial cells when cultured in a high glucose environment.

## Materials & methods

### Cell line

Experiments were performed on the immortalized cell line, rat Müller glial cells (rMC-1), generously obtained from John Mieyal, Case Western Reserve University. Müller cells were exposed to high glucose (25mM) or normal glucose (5mM) Dulbecco’s modified Eagle’s medium (DMEM) (Invitrogen 11995 and Invitrogen 11885, respectively) to mimic hyperglycemic and normal conditions. Both cell culture mediums contained 2% heat inactivated fetal bovine serum and 1% penicillin streptomycin. Cell medium was changed daily to maintain constant glucose load. For mitochondrial assays and oxidative stress assays, Müller cells were grown in a phenol free DMEM normal (5mM) or high glucose media (25mM) of identical composition to phenol-replete medium (Invitrogen 11054–020, 21063–029, respectively). Muller cells were used between passages 14–28 only. Cells were maintained in normal glucose medium for growth and only placed in high glucose medium for treatment.

### Light treatment

**Fig 1:**
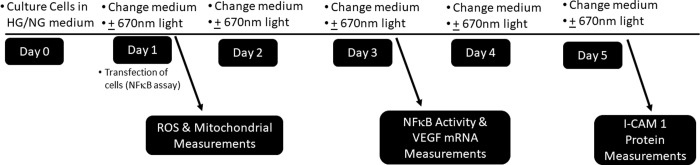
Culture and treatment flow chart. All cells were cultured for experimental on day 0. Cells were treated at each indicated time point and assayed 1 hour after treatment with 670nm light.

Cell cultures were exposed to 670 nm light from a LED array (10 cm x 25 cm) (Quantum Devices Inc. Barneveld, WI) positioned on top of the culture plate at a dose of 25mW/cm^2^ for 180 seconds (total of 4.5J/cm^2^). Sham-treated cells were handled in a similar manner except that the LED array was not illuminated. PBM-treatment occurred one time per day over 24 hours, 72 hours or 96 hours, depending on the assay (pictorially described in Fig 1). All assays were conducted one hour following the final light treatment.

### MTT assay

Cells were plated in triplicate in 96 well plates at a density of 30,000 cells/well, cultured in the respective high or normal glucose media conditions and assayed after 24 hours in culture following light treatment (see Fig 1). After washing with 1xPBS, 5mg/mL MTT was added to cell medium for 3 hours at 37°C. MTT solution was solubilized in 10%Triton x100 and isopropanol with 0.1N HCl (final concentration) for 15 minutes and then read at 590nm (Biotek Synergy HT).

### Assessment of mitochondrial membrane potential

Cells were plated in triplicate in 96 well plates at a density of 30,000 cells/well in normal or high glucose conditions for 24 hours and assayed according to Fig 1. The TMRE (tetramethylrhodamine ethyl ester) assay (Abcam, Cambridge, MA) proceeded per manufacturer’s instructions. Briefly, cells were incubated in 200 nM TMRE dissolved in their respective cell mediums for 30 mins. Cells were washed with PBS and fluorescence (EX/EM: 549/575 nm) was read in 100μL PBS/0.2% BSA solution.

### Assessment of reactive oxygen species

Cells were plated in triplicate in 96 well plates at a density of 30,000 cells/well in normal or high glucose conditions for 24 hours and assayed according to Fig 1. One hour after light treatment, cells were overlain with 40 mM DCFDA (2’7’-dichlorofluorescin diacetate) solution (in respective media), per manufacturer’s instructions (Cayman Chemical Inc., Ann Arbor, MI) and read via fluorescence in a plate reader with EX/EM 484nm/528 nm with a gain of 50–75.

### Transfection of NFκB

Muller cells were plated in a six well dish to confluency of approximately 60–70%. After cells were seated on the plate, they were then co-transfected according to manufacturer instructions with a cocktail containing 1 ml/well Opti-MEM, 5 μl/well lipofectamine 2000, 1 μg/well NFκB plasmid and 0.1 μg/well Renilla plasmid [21]. Transfection was allowed to proceed for 8 hours. Post transfection, the transfection cocktail was aspirated and replaced with cell medium, normal glucose or high glucose, as described above. Cells were treated according to Fig 1. Following treatment, lysates were collected in 1x passive lysis buffer (PLB) and assessed via a Dual Luciferase Reporter assay (Promega catalog number E1910). Luminescence measured was normalized to Renilla. For each replicate, treatment groups were measured in duplicate.

### Assessment of ICAM-1

Cells were cultured and treated according to Fig 1. Cells were harvested and lysed on ice for 15min in 1xRIPA buffer (Alfa Aesar, catalog number J60629) containing 1x protease inhibitor cocktail (Thermo Scientific, catalog number 87786). 100ug of cell lysate boiled at 95°C for 15 min in Laemilli buffer (10% glycerol, 2% SDS, 0.1% bromophenol blue, 200mM Tris HCl pH 6.8, 20mM DTT). Samples were run on an SDS-PAGE gel and transferred to a polyvinyl difluoride (PVDF) membrane. Prior to transfer, the membrane was soaked in methanol for 2 minutes, then washed with Transfer Buffer (0.3%w/v Tris, 1.45% w/v glycine, and 10% v/v methanol). Anti-rabbit ICAM-1(Cell Signaling, catalog number 49155) and anti-rabbit GAPDH (Cell Signaling, catalog number 21185) were used to probe the membrane at 1:500 and 1:5000, respectively. An anti-goat, anti-rabbit horseradish peroxidase-linked secondary antibody (1:10,000, Jackson Labs, catalog number 111-035-144) followed by detection using SuperSignal™ Chemiliuminescent HRP Substrate and exposure to film. ImageJ software was used to compare protein levels normalized to GAPDH.

### VEGF qPCR

Cells were plated in 100mm dishes and treated according to Fig 1. On day 3, cells were washed with 1xPBS and harvest in Trizol. RNA was extracted per manufacturer’s instructions. Biorad iScript cDNA kit was used to create cDNA. qPCR was performed using Biorad SybrGreen Super Mix on a step1plus machine following manufacturers’ instructions. Primers used were for VEGF (FP CCGCAGACGTGTAAATGTTCC, RP GACGGTGACGATGGTGGTGT) and actin (FP: TGACGTGGACATCCGCAAAG, RP: CTGGAAGGTGGACAGCGAGG). Samples were run in triplicate and normalized to the sham, normal glucose control. Melt curve analysis was performed, and data was analyzed by comparing Ct values for sham versus treated VEGF normalized to GAPDH (ΔΔCt).

### Statistical analysis

Samples were measured in duplicate or triplicate. All glucose and light conditions were normalized to the normal glucose sham condition. Averages, standard deviations and standard errors were calculated. Graphs show averages with error bars that represent standard errors of the mean. Differences between each sample group were analyzed by ANOVA, followed by Bonferroni post-hoc testing. The alpha for statistical analysis was set at a p value of 0.05.

## Results

Examination of the effects of 670nm light treatment on signaling pathways that are activated in response to cell exposure to high glucose were performed in an immortalized rat Müller glial cell line (rMC-1). We examined early changes beginning as soon as 24 hours in culture with one treatment of 670nm light and changes occurring within 5 days in culture with 5 treatment procedures (Fig 1). This allowed for examination of the effects of 670nm light in a time dependent manner on both upstream events and downstream events of transcriptional changes due to NFκB activation.

### PBM mitigates ROS produced by high glucose

Oxidative stress is a common and significant factor in DR. Increasing intracellular ROS activates NFκB and other pathogenic mechanisms leading to diabetic retinopathy. Therefore, ROS is expected to increase early in disease. We tested acute exposure of glucose on rMC-1 cells to capture early changes in ROS.

**Fig 2:**
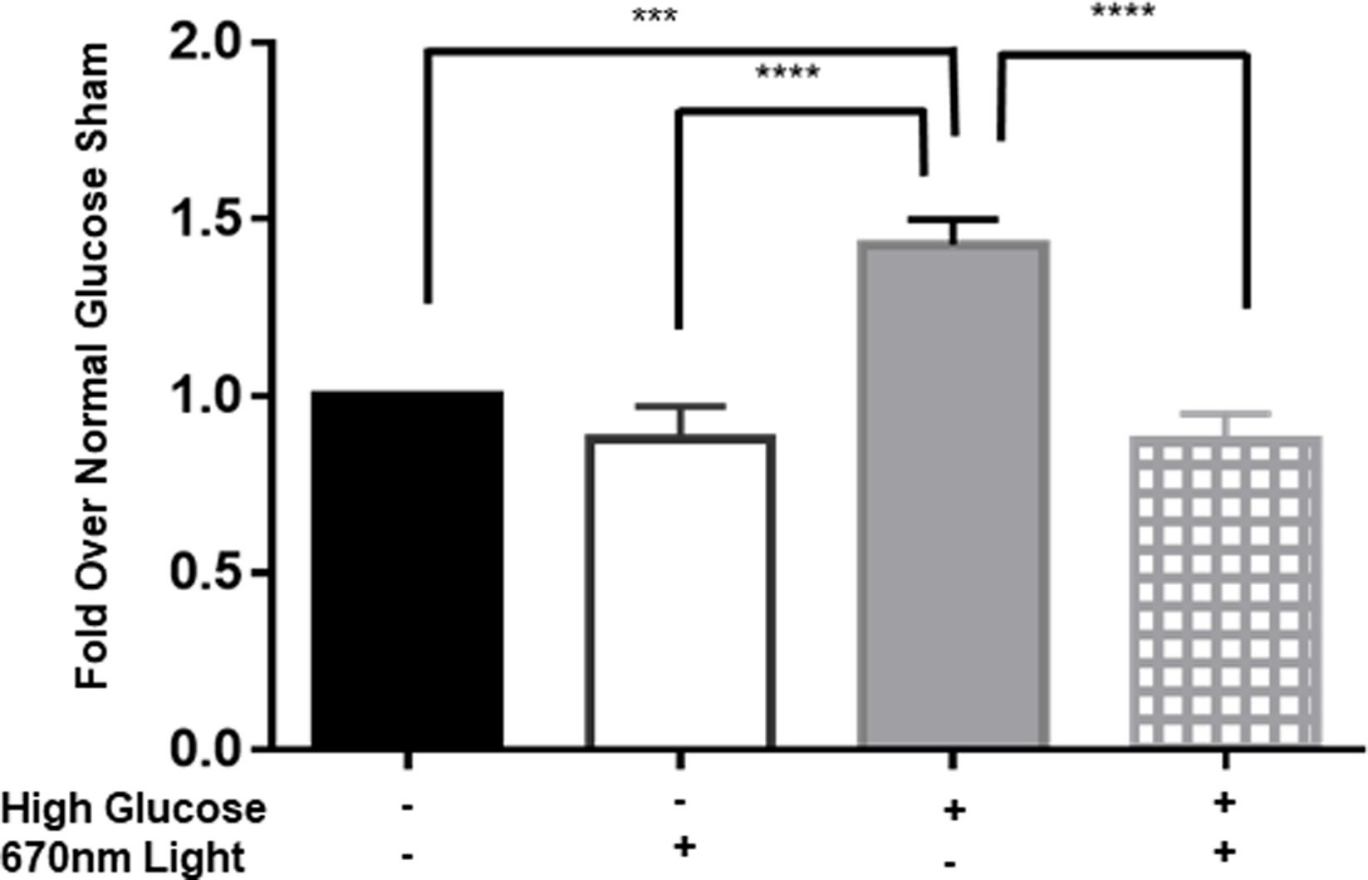
670 nm light treatment diminishes oxidative stress under high glucose conditions in Muller cells. Bar graph displaying ROS production in rMC-1 cells. Cells were cultured in either normal glucose (black bars and white bars) or high glucose (gray bars and hashed bars) medium for 24 hours. 670nm or sham control was applied to the cells at 4.5 J/cm^2^. Oxidative stress was analyzed using DCF-DA. One-way ANOVA statistical analysis yielded *** p<0.001, **** p<0.0001. Three separate experiments were performed in triplicate.

rMC-1 cells acutely exposed to high glucose produced significantly more ROS (50% greater, p<0.001) than those cultured under normal glucose sham conditions (Fig 2). One treatment with 670nm light significantly decreased ROS, to values similar to those measured under normal glucose conditions in rMC-1 cells grown in high glucose for 24 hours. (p<0.0001, compared to high glucose sham). It was not anticipated that PBM would modulate rMC-1 cells exposed to normal glucose conditions due to a lack of stress applied. As expected, 670 nm light did not affect the amount of ROS generated intracellularly.

### Mitochondrial function is diminished with high glucose and partially restored with 670nm light

Because mitochondrial function relies upon healthy mitochondria, we assessed mitochondrial integrity and mitochondrial NADPH-dependent oxidoreductase activity to assess early alterations in mitochondria in response to high glucose.

**Fig 3:**
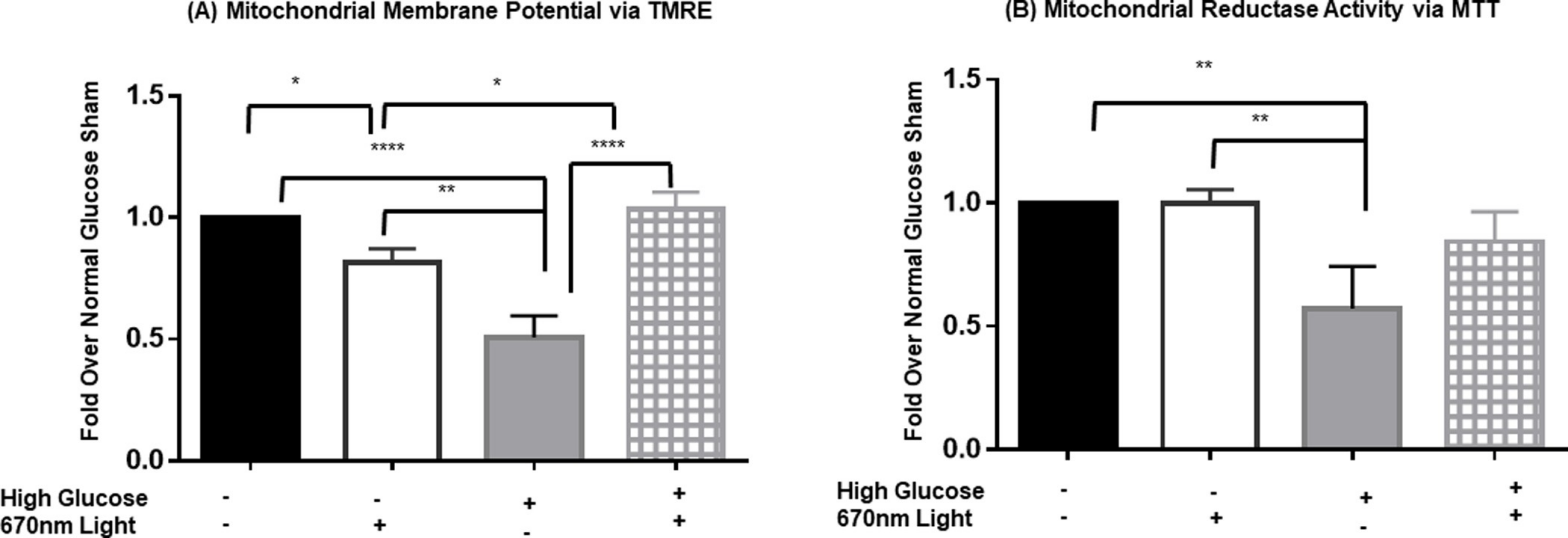
670nm PBM restores mitochondrial function under high glucose conditions. rMC-1 cells were cultured in either normal glucose (black bars and white bars) or high glucose medium (gray bars and hashed bars) for 24 hours. 670nm (white bars and hashed bars) or sham control (black bars and gray bars) was applied to the cells at 4.5 J/cm^2^. All assays were normalized to the normal glucose sham levels. (A) Mitochondrial membrane potential was analyzed using TMRE, (B) NAPDH-dependent oxidoreductase activity was analyzed using MTT. Both TMRE and MTT assays were performed in 3 separate experiments, each data point was measured in triplicate. One-way ANOVA analysis yielded statistics as follows: * p<0.05, ** p<0.01, **** p<0.0001.

Mitochondrial membrane potential (MMP), a reliable indicator of mitochondrial integrity and function, can impact ATP production and oxidative stress [37]. We assessed MMP via TMRE assay where a decrease in TMRE fluorescence indicates a reduction in MMP and disrupted mitochondrial energy production. rMC-1 cells in high glucose conditions for 24 hours and treated with 670 nm light showed significantly increased TMRE values compared to the high glucose sham group (Fig 3A). This increase shows the restoration of mitochondrial membrane potential in addition to improved mitochondrial function, indirectly. At the same time, the high glucose 670 nm light-treated rMC-1 cells showed no significant difference from the normal glucose sham group (Fig 3A).

We also examined NADPH-dependent oxidoreductase activity via MTT assay. While commonly used to measure cell viability, MTT specifically measures the function of the mitochondrial reductase and alterations in MTT signal are indicative of cellular stress in the absence of cell death [38]. Thus, we examined early changes in rMC-1 cells in response to high glucose medium with and without 670nm light treatment. rMC-1 cell NADPH-dependent oxidoreductase activity was reduced by nearly 50% following 24 hours in high glucose medium, indicative of a reduction in mitochondrial respiration and cellular energy capacity. A single treatment with 670 nm light (dose 4.5 J/cm2) increased NADPH-dependent oxidoreductase activity (Fig 3B) by 60% from high glucose conditions, to values similar to those measured in cells cultured in normal glucose concentrations. In addition, we examined cellular ATP content, however we did not observe any differences likely due to the early time point assessed and the glycolytic nature of these cells [39].

### High glucose causes transcriptional changes of NFκB and leads to increased production of ICAM-1

**Fig 4:**
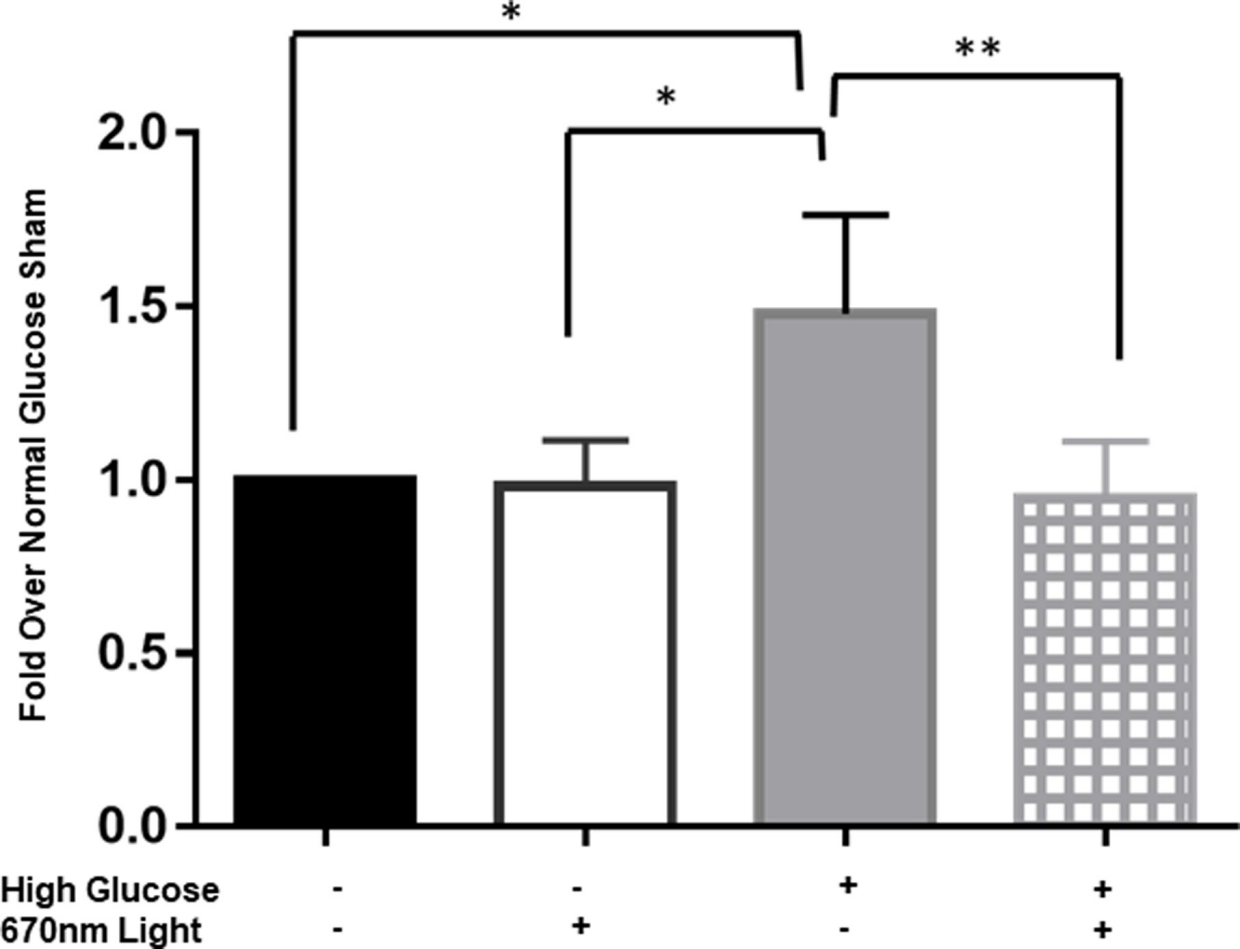
670nm light treatment diminishes NFκB activity under high glucose conditions in Muller cells. Bar graph showing Müller cells were cultured in either normal glucose (black bars and white bars) or high glucose medium (gray bars and hashed bars) for 72 hours while transfected with NFκB-luciferase. NFκB activity was analyzed via reporter gene assay, normalized to the transfection control, Renilla, and then treatments were normalized to normal glucose, sham conditions. 670nm (white bars and hashed bars) or sham control (black bars and gray bars) was applied to the cells at 4.5 J/cm^2^ daily. One-way ANOVA statistical analysis yielded *p>0.05, ** p<0.01 Four separate experiments were performed in duplicate.

With early changes in ROS and mitochondrial function, we next looked at downstream transcriptional changes associated with high glucose. The transcription factor, NFκB, is modulated by ROS levels in high glucose conditions [4, 16, 40] to lead to inflammatory protein transcription. We examined NFκB in our system through a luciferase reporter assay. When rMC-1 cells were cultured in high glucose, there was a significant increase in NFκB activity in comparison to cells cultured in normal glucose. As shown in Fig 4, a 50% increase in luminescence in the high glucose cultured groups was observed in comparison to those cultured in normal glucose. NFκB activity was reduced to that observed in normal glucose conditions when treated with 670 nm PBM. This reduction was significantly different that high glucose/sham treated samples. There was no statistical difference between the level of NFκB activation in the normal glucose group receiving sham treatment in comparison to the high glucose group receiving PBM treatment. These results indicate that PBM entirely attenuated hyperglycemia induced activation of NFκB after three days in culture.

**Fig 5:**
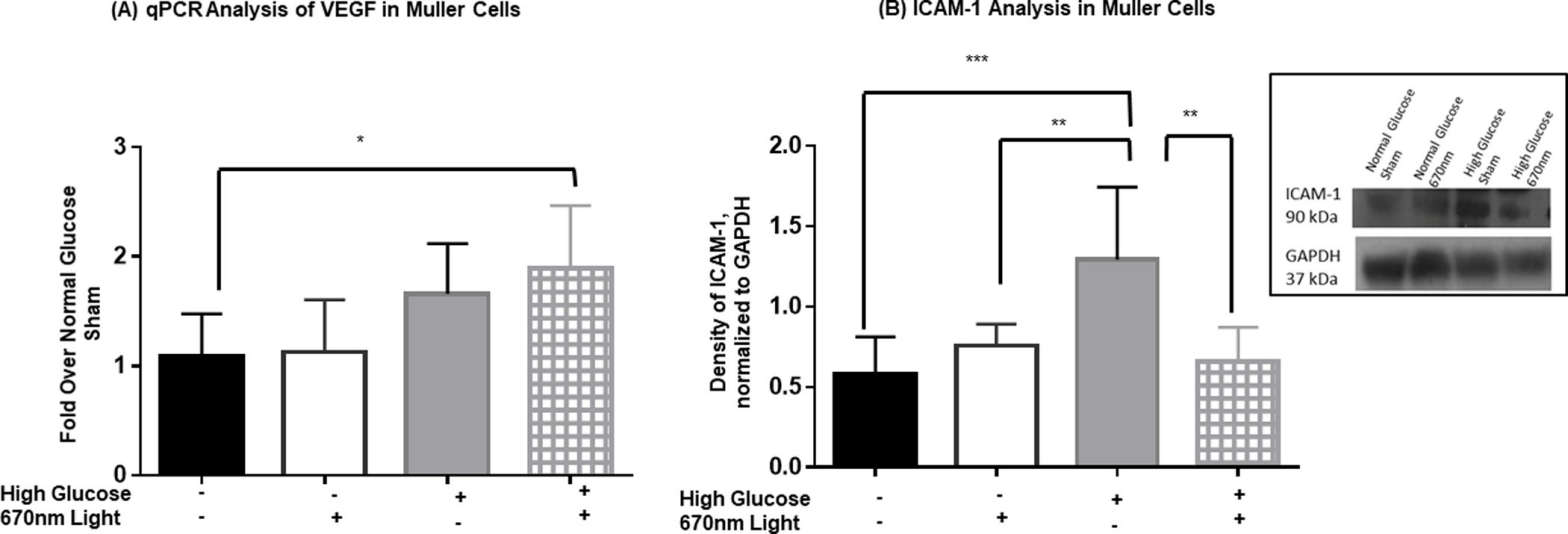
670nm PBM suppresses downstream mediators of NFκB signaling. Bar graph representing rMC-1 cells were cultured in either normal glucose (black bars and white bars) or high glucose (gray bars and hashed bars) medium for 72 hours. 670nm (white bars and hashed bars) or sham control (black bars and gray bars) was applied to the cells at 4.5 J/cm2 daily while in culture. (A) VEGF mRNA was analyzed via qPCR and normalized to actin. mRNA levels were calculated by ΔΔCt and normalized to Normal glucose sham. Five separate experiments were performed in duplicate. One-way ANOVA statistical analysis yielded * p = 0.0147 (B) ICAM was analyzed via western blot and normalized to GAPDH as the loading control via ImageJ. Inset includes a representative western blot image. Six separate experiments were performed, and one-way ANOVA analysis yielded * p<0.05, ** p<0.01, *** p<0.001.

In response to high glucose, NFκB becomes activated and upregulates transcription of a variety of pro-inflammatory molecules and other molecules associated with angiogenesis and adhesion [41]. Thus, we then examined VEGF via qPCR to determine if hyperglycemia altered VEGF in rMC-1 cells. Following 3 days in hyperglycemic culture conditions, VEGF transcripts were 50% larger than VEGF levels under normal glucose conditions (Fig 5A). We chose this earlier time point since we were looking at mRNA levels of VEGF and we expect those to change in a similar time frame as transcriptional activation of NFκB. Unexpectedly, high glucose, PBM treated Müller glial cells did not show decreased levels of VEGF transcript expression in comparison to high glucose sham treated conditions. This could be due to regulation of VEGF levels by other factors in addition to NFκB, see Discussion.

Lastly, we measured ICAM-1 concentrations by western blot in cell lysates from Müller cells cultured in either high or normal glucose medium. We maintained cells in culture for 5 total days. This is longer than the time frame used for NFκB activity analysis to provide time for gene transcription and protein translation to occur. As shown in Fig 5B, we measured an increase in ICAM-1 concentrations in cell lysates via western blot from cells cultured in high glucose compared to those cultured in normal glucose. ICAM-1 levels doubled in cells cultured in high glucose compared to cells grown in normal glucose. Likewise, 670nm light treated cells showed reduced ICAM-1 levels, similar to that seen in the normal glucose controls.

## Discussion

The goal of this study was to enhance understanding of the modulation of signaling pathways by 670nm light, characterizing early changes as well as those occurring after an acute exposure of high glucose over a period of days. Our studies showed that as little as 24 hours of high glucose exposure is enough to disrupt mitochondrial function and increase ROS production in rMC-1 cells. These early ROS and mitochondrial function changes initiate a cascade of signaling events leading to NFκB activation and increased ICAM-1 and VEGF production. The changes were mitigated by treatment with 670nm light which attenuated ROS production within an hour and improved MMP. Daily, short treatment with 670nm light continued to mitigate detrimental signaling effects seen with high glucose alone including reduced NFkB transcriptional activity and decreased production of ICAM-1. Interestingly, we saw no light treatment benefit on the production of VEGF implicating its production by another pathway in addition or in lieu of NFκB. While our study has been limited by examination of PBM effects within the immortalized cell model of early DR, these findings are consistent with other model systems and pathways in which PBM has been shown to be beneficial [10, 16].

Oxidative stress has been implicated as an initiating factor in DR pathogenesis [7, 21, 32, 33, 42]. In cell models of DR, oxidative stress is increased in multiple cell types including human Müller glial cells treated with tert-butyl hydroperoxide [43], retinal pigment epithelial cells grown in high glucose, and retinal ganglion cells grown in high glucose [13]. In our study, ROS increases in 24 hours of high glucose treatment. Novel to the current study, we report this increase in ROS is mitigated in one hour by a single, short light treatment (670nm, 180s). These findings are consistent with others where PBM has been shown to attenuate oxidative stress in many disease models, including DR [9, 10, 12–14, 44]. However, unlike the current study, the majority of these studies have investigated the effects of 670 nm PBM on oxidative stress and ROS production several days after exposure to cytotoxic stressors including high glucose.

Increased oxidative stress can lead to mitochondrial changes. In our study, we observed loss of MMP in as little as 24 hours in high glucose suggesting this is an early event in response to elevated glucose levels. Although, we did not investigate the order of insult, our study is consistent with Zhang *et al*. who report a mild oxidative stress (100μM H_2_O_2_) leads to a reduction of MMP in human Muller cells [43]. In addition, the current study supports the proposed mechanism of low level light therapy with nitric oxide dissociation to increase the MMP [10, 45, 46].

We observed an increase in NFκB activity at 3 days in culture, likely due to the increased ROS and decreased mitochondrial health. In our study, a 50% increase in NFκB transcriptional activity in the high glucose cultured, sham treated groups was observed in comparison to cells cultured in normal glucose and not receiving treatment (Fig 2). These findings are consistent with previous work [21, 47] where increased NFκB activity was observed in response to increased oxidative stress through high glucose in Müller glial cells [21], in monocytes (THP-1) [48] and pericytes [49]. In streptozotocin-induced diabetic rats, activation of NFκB was increased by 60% in retinal samples compared to non-diabetic counterparts [50]. Corresponding to this increase in NFκB activity was a reduction in the antioxidant capacity of superoxide dismutase and an increase in oxidative stress, as measured by the presence of 8-isoprostane in the blood and ratio of GSSG/GSH. These changes were partially attenuated with treatment of the antioxidant, resveratrol [50].

Importantly, NFκB concentrations in PBM-treated high-glucose-exposed cultures were not different from that measured in cells maintained under normal glucose. We believe this attenuation is likely due to the decreased ROS and early prevention of mitochondrial changes. These findings are consistent with other studies examining PBM and supporting the purported mechanism [10, 51–54].

NFκB is a transcription factor that regulates many types of genes that are involved in many cellular functions including inflammation, cell adhesion, growth, stress responses [13, 21, 42]. Pertinent to this study, we examined downstream targets of NFκB implicated in DR pathogenesis, ICAM-1 and VEGF. Elevated levels of ICAM-1 have been associated with other in vitro [21] and in vivo [12, 14, 55] models of DR. As expected, we observed a doubling of ICAM-1 with high glucose conditions, similar to the increase observed in NFκB activity, and consistent with other reports [21]. This increased production was mitigated with PBM. Our *in vitro* data on the effects of PBM on ICAM-1 concentrations in high glucose-cultured Müller glial cells was comparable to the *in vivo* effects of PBM in diabetic rodents [12, 13]. Since ICAM-1 is highly regulated by NFκB, it is likely that for the observed decrease in NFκB transcriptional activity there will be a corresponding decrease in ICAM-1 level [56–58].

Also, VEGF levels increased with high glucose treatment, consistent with many other reports [22, 25, 34, 40]. Unexpectedly, we observed no change in VEGF mRNA levels under high glucose conditions compared to high glucose cultured cells treated with 670nm light. This data indicates that in this cell culture model of DR, VEGF and ICAM-1 are not regulated in the same manner. Regulation of VEGF can occur through multiple pathways in addition to NFκB including, p38, TNFα, CREB, vPA, IL-1B, Era, Stat3, and HCAM [40], and these pathways may not be altered with 670nm light treatment.

In summary, our findings of the acute exposure of immortalized rMC-1 cells to high glucose show early changes in ROS production and mitochondrial decline, both of which are mitigated with 670nm treatment. Prolonged high glucose culture conditions also lead to NFκB activation and ICAM-1 production. These changes are prevented with 670nm light treatment. Thus, 670nm light can prevent the acute effects of high glucose in an early DR immortalized cellular model system and reduce the production of pro-inflammatory mediators, providing a mechanism by which PBM is beneficial.

## Supporting information

S1 File Raw image files of western blot for ICAM and GAPDH. Cropped, representative image is seen in Fig 5. Each image is captioned to denote loaded sample and location of protein of interest.
(PDF)
