# Supplementary material for: 670nm photobiomodulation modulates bioenergetics and oxidative stress, in rat Müller cells challenged with high glucose
Source: PLoS One. 2021 Dec 3;16(12):e0260968. doi: 10.1371/journal.pone.0260968 (PMC8641888; doi:10.1371/journal.pone.0260968)
Supplement: S1 File — Cropped, representative image is seen in Fig 5. Each image is captioned to denote loaded sample and location of protein of interest. (PDF) [file pone.0260968.s001.pdf]

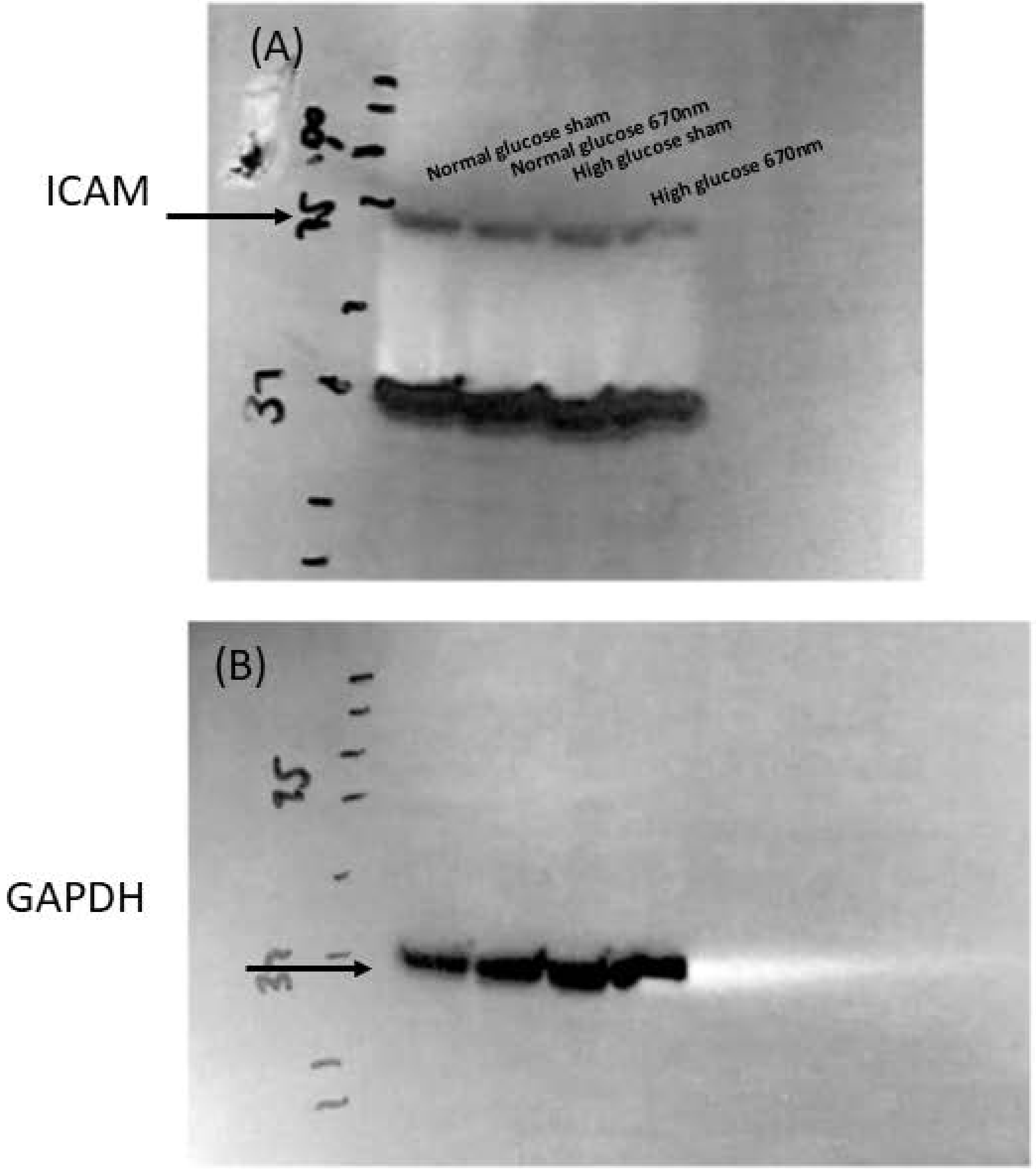

Figure S1: Full length western blot image for ICAM and GAPDH. Cropped image is seen in Figure 4. (A): ICAM reactivity shown as ladder, normal glucose sham, normal glucose 670nm light, high glucose sham, high glucose 670nm light. (B) GAPDH reactivity shown as ladder, normal glucose sham, normal glucose 670nm light, high glucose sham, high glucose 670nm light. Arrow denotes band of interest.

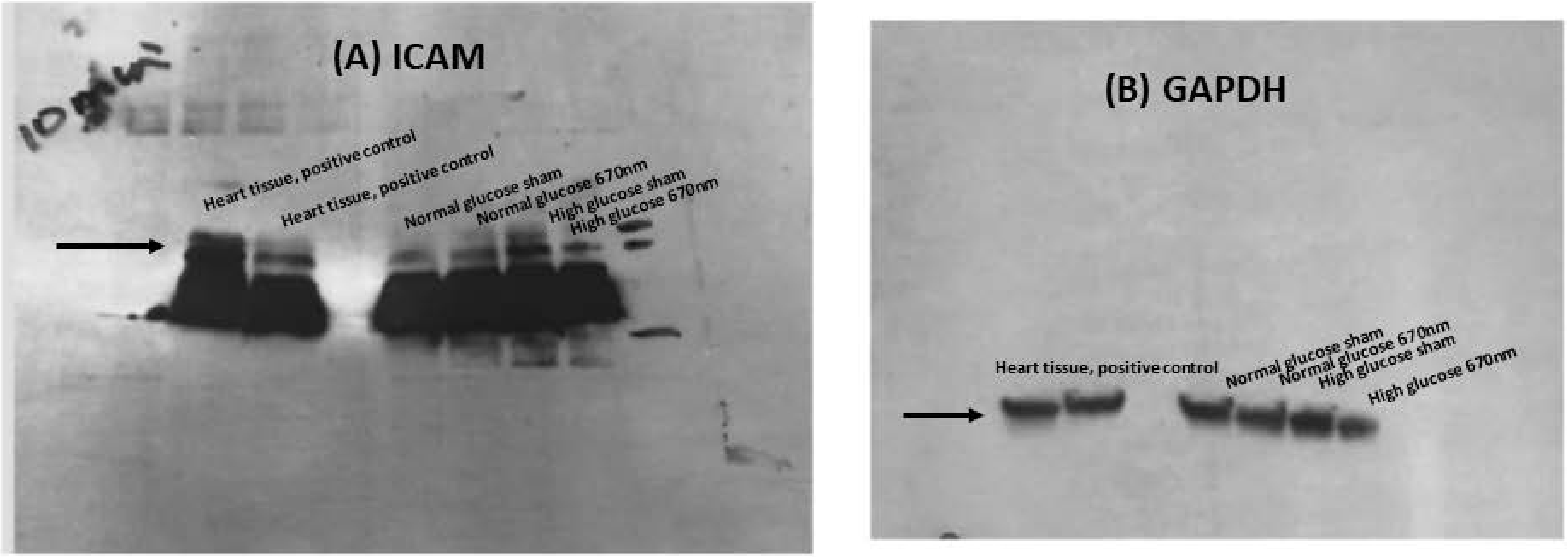

Figure S2: Full length western blot image for ICAM and GAPDH. Cropped image is seen in Figure 4. (A): ICAM reactivity shown as positive control (heart tissue, mouse), positive control (heart tissue, mouse), empty well, normal glucose sham, normal glucose 670nm light, high glucose sham, high glucose 670nm light, ladder. (B) GAPDH reactivity shown as positive control (heart tissue, mouse), positive control (heart tissue, mouse), empty well, normal glucose sham, normal glucose 670nm light, high glucose sham, high glucose 670nm light, ladder. Arrow denotes band of interest.

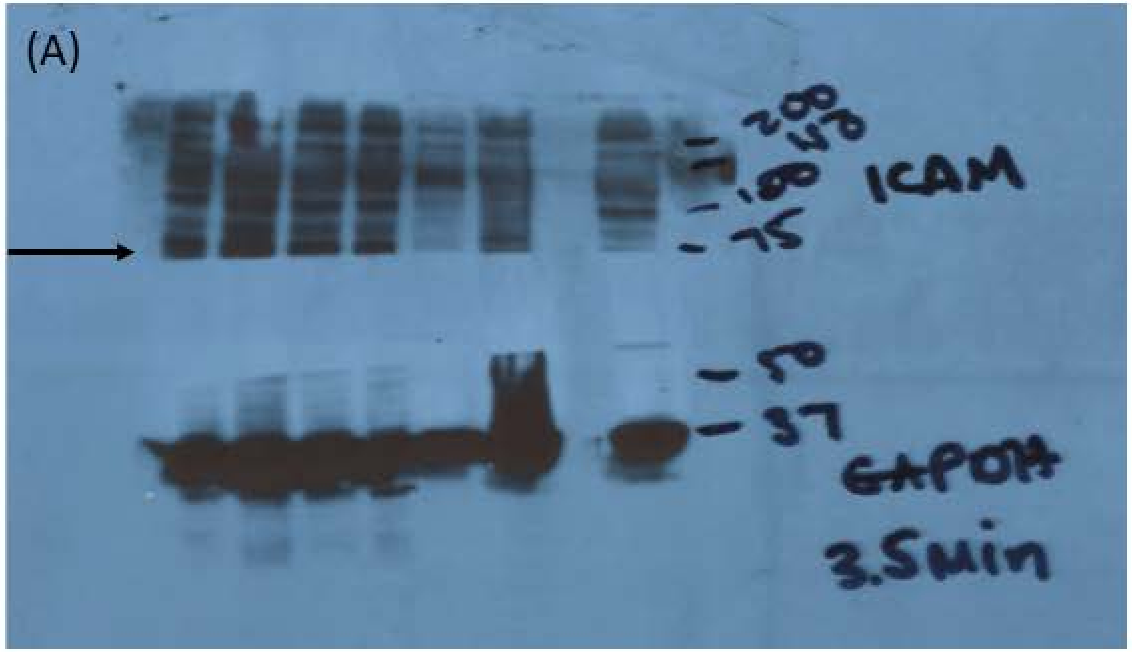

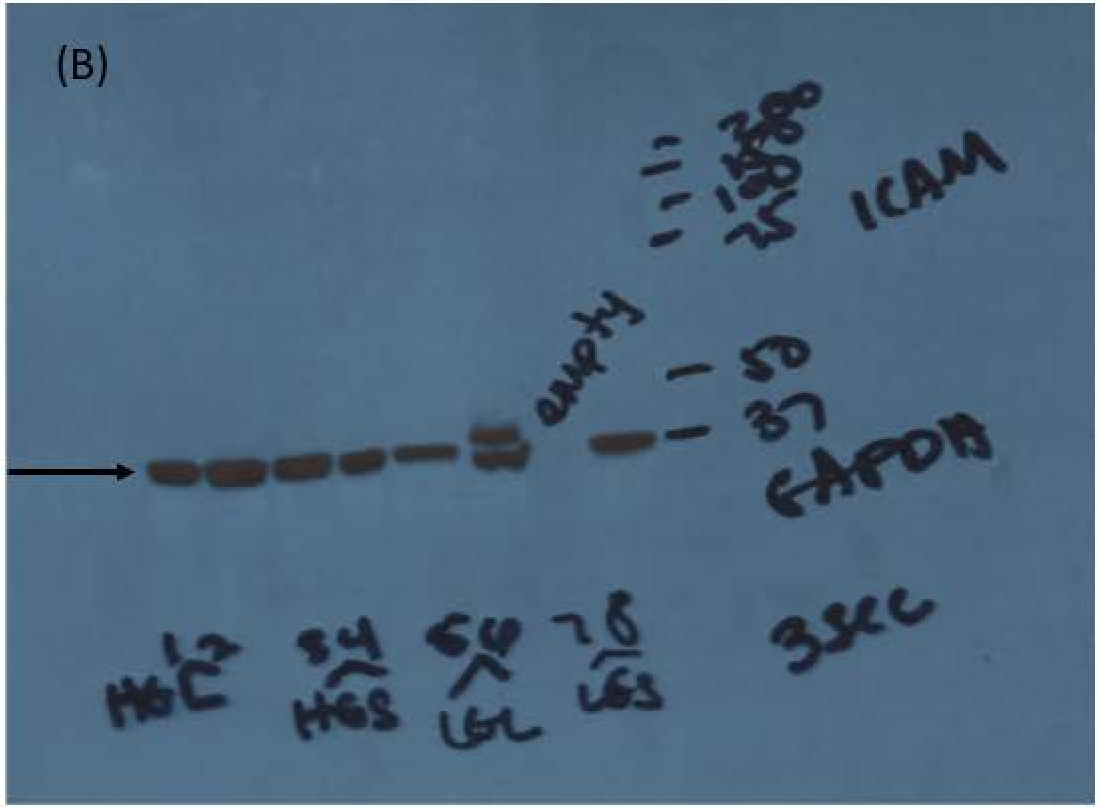

Key:
HGL=high glucose, 670nm
HGS=high glucose, sham
LGL=low glucose, 670nm
LGS=low glucose, sham

Figure S3: Full length western blot image for ICAM and GAPDH. Cropped image is seen in Figure 4. (A): ICAM reactivity shown as high glucose 670nm light, high glucose 670nm light, high glucose sham, high glucose sham, normal glucose 670nm light, normal glucose 670nm light, empty, normal glucose sham, ladder. (B) GAPDH reactivity shown as high glucose 670nm light, high glucose 670nm light, high glucose sham, high glucose sham, normal glucose 670nm light, normal glucose 670nm light, empty, normal glucose sham, ladder. Each sample represents a single replicate (i.e. 2 independent replicates of high glucose 670 nm). Arrow denotes band of interest.
